# An intuitive Python interface for Bioconductor libraries demonstrates the utility of language translators

**DOI:** 10.1186/1471-2105-11-S12-S11

**Published:** 2010-12-21

**Authors:** Laurent Gautier

**Affiliations:** 1 DMAC, Centre for Biological Sequence Analysis, Department of Systems Biology, Technical University of Denmark, Matematiktorvet, 2100 Lyngby, Denmark

## Abstract

**Background:**

Computer languages can be domain-related, and in the case of multidisciplinary projects, knowledge of several languages will be needed in order to quickly implements ideas. Moreover, each computer language has relative strong points, making some languages better suited than others for a given task to be implemented. The Bioconductor project, based on the R language, has become a reference for the numerical processing and statistical analysis of data coming from high-throughput biological assays, providing a rich selection of methods and algorithms to the research community. At the same time, Python has matured as a rich and reliable language for the agile development of prototypes or final implementations, as well as for handling large data sets.

**Results:**

The data structures and functions from Bioconductor can be exposed to Python as a regular library. This allows a fully transparent and native use of Bioconductor from Python, without one having to know the R language and with only a small community of *translators* required to know both. To demonstrate this, we have implemented such Python representations for key infrastructure packages in Bioconductor, letting a Python programmer handle annotation data, microarray data, and next-generation sequencing data.

**Conclusions:**

Bioconductor is now not solely reserved to R users. Building a Python application using Bioconductor functionality can be done just like if Bioconductor was a Python package. Moreover, similar principles can be applied to other languages and libraries. Our Python package is available at: http://pypi.python.org/pypi/rpy2-bioconductor-extensions/

## Background

The Bioconductor project [1], based on the R language [2], has become a reference for the numerical processing and statistical analysis of data coming from high-throughput biological assays. Starting with microarray data, it became an integrated suite of data structures and functions to perform tasks ranging from reading raw data files to processing algorithms and to data analysis. The project soon expanded to data analysis in bioinformatics at large and to other assays, providing a rich selection of methods and algorithms to the research community.

At the same time, the Python language [3] has matured as a dependable platform for prototype development and data handling. Python is used by many organizations in need of processing or analyzing large volumes of data (Google, NASA, CERN, ILM). Python is a very accessible language and is used in introductory courses to programming for non-computer scientists [4,5]. It is also used by professional programmers in need of increased productivity [6] and agile prototyping.

In the context of bioinformatics, the Biopython project [7] was one of the first Python libraries for bioinformatics, and while a few utilities offered by the Bioconductor project were ported to it, both projects grew independently. A collection of other bioinformatics-related Python libraries has also appeared during the last few years: PyCogent [8], pygr [9], and bx-python [10], to name a few.

We choose the R/Bioconductor-Python duo in the context of bioinformatics to demonstrate how bridging software libraries in different languages can be performed. There exists other bioinformatics libraries in other languages [11-14] with which similar principles could be applied, given the relevant tools for bridging the different languages.

## Results and discussion

### Communities and translation

Whenever a project spans across several communities, the issue of language arises. Bioinformatics is an example of that: being at the interface between biology, computer science, information technology, and statistics, it requires translating terms when experts in the different fields communicate. Here we are focusing on computer languages but the very same principles apply to disciplines. The analogy is even more appropriate when the practitioners of the different disciplines favor one computer language over another one.

Having a bilingual community is a good way to make cross-language barriers fall, but it has the substantial drawback of being relatively difficult and expensive to achieve. When hiring technical specialists, finding experts in a field can be a difficult task, let alone experts in two fields. Moreover, requiring a bilingual community to operate could cause insidious problems: the imperfect mastery of at least one of the two computer languages can help create issues and keep them unnoticed.

A smaller community of bilingual individuals, we shall call *translators* or *interpreters,* is able to bridge two larger communities and is easier to obtain than a bilingual community even when setting high standards of fluency for both languages. *Translators* can be in charge of exposing written blocks in one language, which are here Bioconductor data structures and functions written in R, into meaningful blocks in another language, here Python. The result is an interface layer that can be used without knowing much of the original language in which the libraries were developed.

The software package presented here demonstrates that a translation layer can provide Python developers access to the Bioconductor project, and allow them to develop applications without knowing R.

#### Exposing Bioconductor/R structures as native Python structures

The role of *translators/interpreters* can be restricted to wrapping Bioconductor libraries as Python classes. Here we propose to expose Bioconductor to a Python user, and we rely on the Python-to-R bridge *rpy2*[15]. This bridge embeds an R interpreter into a Python process and allows seamless access to R objects and functions. This bridge removes the need to deal with the technical issues related to accessing R from Python and lets us focus on presenting Bioconductor libraries to Python programmers.

**Figure 1:**
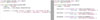
**Implementing a Bioconductor class in Python**. Implementing a Bioconductor class can be performed by either declaring all class components explicitly (right), or by relying on meta-programming utilities found in the *rpy2* package (left). Meta-programming helps reduce the amount of boilerplate code by automating part of the translation work.

In essence, Bioconductor packages contain functions, data structure definitions (classes), and data objects (instances). The task of *translators* is to represent these in Python. This can be done manually, or semi-automatically when relying on the meta-programming tools found in the *rpy2* package (See Figure 1). Functions usually do not need much work as they are already automatically exposed by *rpy2.*

The object system in Python is fairly unified, despite the remaining existence of *old* and *new* objects in the Python 2.x series, and is very much central to the language. Most, if not all, Python programmers will be familiar with it. In contrast, the Bioconductor project makes extensive use of the S4 class system for R, a system that remains less known to many R users. The S4 system is related to the one of Common Lisp Object System (CLOS) [16], and offers multiple dispatch for methods. The S4 system is only present in a limited number of languages (beside CLOS, Clojure’s *multimethods* can be mentioned [17]), and is not available in Python. In this context, the difference in object-oriented programming paradigms have to be resolved by *translators/interpreters.*

Rpy2 exposes classes and methods from Bioconductor are exposed in such a way that differences in programming languages are alleviated. The resulting overall structure matches the canons of Python programming, which Python programmers refer to as *being Pythonic.* The translation proposed creates Python classes corresponding to the Bioconductor classes, and creates Python methods for the relevant S4 methods. The class and method names are kept across the translation, with minor exceptions for methods. Suffixes are added to the method name when S4 multiple dispatch results in naming conflicts on the Python side, and in that case, the type of the arguments in the signature are added to the method names. For example, the *biostrings* class *PairwiseAlignedXStringSet* has three static methods *fromXString_XString(), fromCharacter_Character(), fromCharacter_missing()* to represent the three corresponding constructors of *PairwiseAlignedXStringSet* in Bioconductor. This approach helps keeping a high ressemblance between Python and Bioconductor for the functionalities translated.

**Figure 2:**
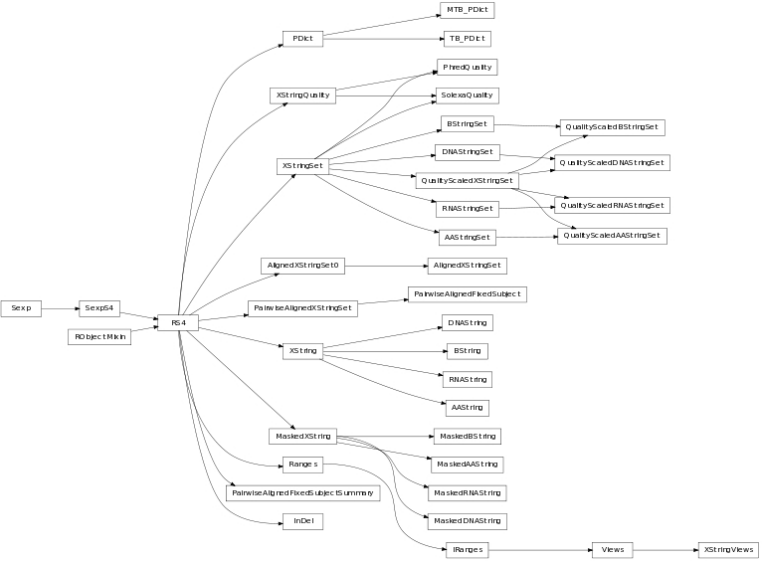
**Class diagram for the translation of *Biostrings***. Class from the Bioconductor package *Biostrings* are exposed to Python as native classes. Parent-child relationships are represented by arrows.

Bioconductor packages can define numerous classes, so it is important that the task of exposing them to Python programmers remains as simple and as short as possible. The Bioconductor package *Biostrings* alone contains close to 40 classes exposed to Python, as illustrated Figure 2, while the code base for the translation remains of relatively modest size: *Biostrings* is exposed in less than 600 lines of code, so less than 15 lines of Python code per class exposed on average. In our implementation Python classes are essentially wrappers for R methods of Bioconductor classes, limiting the need for extensive testing.

In addition to the above, the task of the *translators/interpreters* can go beyond exposing the classes. Translating idioms specific to one language into the other language will increase the quality of the translation (for example Python has iterators, not available by default in R and not used in Bioconductor). *Translators* can also present data structures a different way, and build a new API from the existing Bioconductor libraries. This is of interest in the context of different communities with different views on data structures and methods, as one can quickly rewrap the existing libraries. This can also be helpful for hiding sophisticated options and simplifying the interface, or wrapping sequences of function calls.

The implementation presented here covers several Bioconductor infrastructure packages, and is sufficient to handle annotation data, genome sequences, microarray data, and next-generation sequencing.

**annotationdbi:** infrastructure for handling biological annotations.

**biobase:** infrastructure for handling data from high-throughput assays.

**biostrings:** infrastructure for handling biological strings (DNA, RNA, protein sequences)

**bsgenome:** infrastructure for handling genome sequences

**edger:** differential digital expression data

**geoquery:** query data resources from the Gene Expression Omnibus (GEO) repository.

**ggbase:** infrastructure for genetics of gene expression

**ggtools:** software and data for genetics of gene expression

**goseq:** Gene Ontology analysis for RNAseq

**gseabase:** infrastructure for Gene Set Enrichment Analysis (GSEA) types of methods

**iranges:** infrastructure for handling interval data

**shortread:** infrastructure for handling datasets of short reads

### Case-study: providing a web-based interface to *edgeR*

The *egdeR* method [18] is a popular statistical method for measuring differential abundance in RNA molecule when the measurement technology is based on counts. It is useful for SAGE and RNAseq data. Having the method easily accessible to a community outside the regular Bioconductor user-base expands its reach to the scientific community. In this scenario a simple web application is considered, and the application is written in Python. One strong advantage of Python over R is the presence of many industry-grade solutions for developing web applications, and we choose to demonstrate how to build such a application with *edgeR.*

#### Reproducing R code

**Figure 3:**
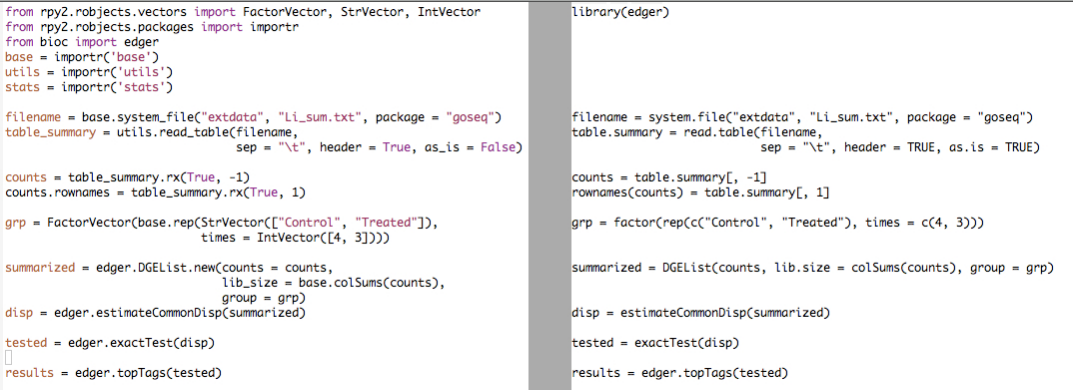
**Case study: *edgeR* with Python**. The Python code (left) differs from the R code (right) mostly in the top part, where the Python code is using explicit import procedures.

The Bioconductor/R *edgeR* library is exposed to Python in the module *bioc. edger,* and following the documentation written for R users is straightforward (See Figure 3). As outlined earlier, classes and methods present in the Bioconductor package are represented by matching Python classes and methods, as the *translator/interpreter* focused on keeping a high resemblance between R and Python code.

#### Building a prototype web server

**Figure 4:**
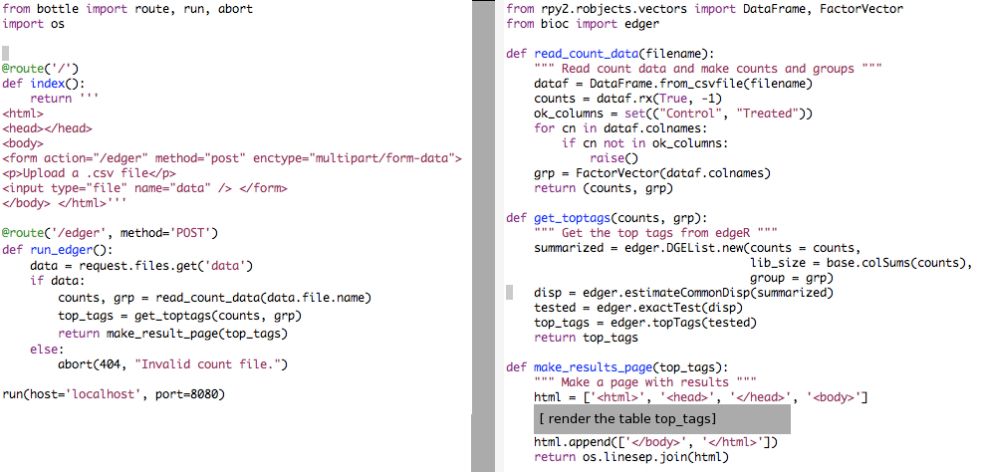
**A full webserver for *edger***. In this scenario Python developers have to develop quickly a prototype website letting users upload a CSV file of gene counts and obtain the table of top differentially counted genes. The main application is minimal (left), relying on three helper functions derived from the earlier example with running *edgeR* (right). Rendering the result table is not detailed in order to keep the example concise.

The code used to perform an *edgeR* analysis can be wrapped by the Python developer into a Python function, and building a web application that calls this function is trivial (See Figure 4).

A fully functioning self-sufficient prototype, including a web-server, a web-form to upload data, data processing, computation of results from the data uploaded, and an answer returned to a client web browser, can be implemented in less than 100 lines of code.

Having the web server implemented in Python is deemed better because Python has a strong track record of agile web frameworks, the language possesses better error handling mechanisms, and it allows a decoupling of the implementation of data analysis (in R) from the implementation of the application. This separation is important since it allows a programmer specialized in the development of web applications to utilize code developed in R/Bioconductor by data analysts. The translation layer ensures that the code in Bioconductor is exposed in such a way that it can be integrated into the application while retaining all the benefits of the host language.

This example emphasizes the ease with which applications can be built, and relies on a minimal web development framework. There exist more comprehensive and more complex frameworks, such as Django [19] and Plone [20]. Similar implementations have been performed with them. In these cases the development of applications requires highly specialized skills in the corresponding frameworks. In a context where there is specialization of people because of increasingly complex domain-specific knowledge, the availability of a translation layer such as the one proposed is crucial: data analysts can therefore focus on developing algorithms while application developers can focus on the application.

## Conclusions

A relatively small community of people fluent in two languages and disciplines can expose data structure definitions and functions from libraries in one language as code directly usable by practitioners of the other language. We demonstrate here how this can be achieved by creating a bridge from the Bioconductor project, a popular set of R libraries for the analysis of bioinformatics data, to the Python language. Work that requires extensive knowledge of both languages can be restricted to a small community of *translators/interpreters,* and their code be used by Python programmers without the knowledge of R or Bioconductor. The implementation presented here shows that the amount of translation work can be minimal, yet enable the development of Python applications using Bioconductor easily. Our implementation covers key infrastructure packages in the Bioconductor project and can constitute a basis for extending this to more packages in Bioconductor.

As an example we demonstrated how a complete web application computing differential expression for digital gene expression can be implemented.

## List of abbreviations

**CERN:** Conseil Européen pour la Recherche Nucléaire; **CLOS:** Common Lisp Object System; **DNA:** Desoxyribonucleic Acid; **GEO:** Gene Expression Omnibus; **GSEA:** Gene Set Enrichment Analysis; **ILM:** Industrial Light and Magic; **NASA:** National Aeronautics and Space Administration; **RNA:** Ribonucleic Acid; **RNAseq:** Whole Transcriptome Shotgun Sequencing; **SAGE:** Serial Analysis of Gene Expression

## Competing interests

The author declares he has no competing interests

## Methods

### Software and operating systems

#### R/ Bioconductor

The principles detailed here were applied to Bioconductor Release 2.6 (April 2010). Bioconductor packages evolve quickly and new versions do not always maintain backward compatibility. Minor adaption might be necessary in order to run what is presented here with other releases. The Bioconductor release 2.6 requires R-2.11, available on the project’s website [21].

#### Python and libraries

Python 2.6.4 was mainly used for development. Other version in the 2.6 series will work. Python is available with most Linux distribution, and is shipped with OS X Leopard and Snow Leopard (version 2.5 and 2.6 respectively).

A development snapshot of the *rpy2*[15] package (2.2-dev) was used in this work. Minor adaptations will be required for it to work with the current rpy2 release 2.1.

The lightweight web-framework *bottle*[22] was used to demonstrate the implementation of a web-based interface.

#### Operating system

The solution was developed and tested under both Ubuntu Linux 10.04 and 10.10 [23] and Apple OS X Leopard.

## Authors contributions

LG designed and implemented the software, and wrote the manuscript.
